# Gesture-Based Physical Stability Classification and Rehabilitation System

**DOI:** 10.3390/s25196098

**Published:** 2025-10-03

**Authors:** Sherif Tolba, Hazem Raafat, A. S. Tolba

**Affiliations:** 1 Independent Researcher, Franklin, MA 02038, USA; sherif.tolba@uconn.edu; 2 Computer Science Department, Kuwait University, Kuwait City 13060, Kuwait; 3 Faculty of Computer and Information, Mansoura University, Mansoura 35516, Egypt; astolba@mans.edu.eg

**Keywords:** gesture recognition, physical stability, rehabilitation, DFRobot gesture and touch sensor, microcontroller, gesture analysis, deep learning, machine learning

## Abstract

This paper introduces the Gesture-Based Physical Stability Classification and Rehabilitation System (GPSCRS), a low-cost, non-invasive solution for evaluating physical stability using an Arduino microcontroller and the DFRobot Gesture and Touch sensor. The system quantifies movement smoothness, consistency, and speed by analyzing “up” and “down” hand gestures over a fixed period, generating a Physical Stability Index (PSI) as a single metric to represent an individual’s stability. The system focuses on a temporal analysis of gesture patterns while incorporating placeholders for speed scores to demonstrate its potential for a comprehensive stability assessment. The performance of various machine learning and deep learning models for gesture-based classification is evaluated, with neural network architectures such as Transformer, CNN, and KAN achieving perfect scores in recall, accuracy, precision, and F1-score. Traditional machine learning models such as XGBoost show strong results, offering a balance between computational efficiency and accuracy. The choice of model depends on specific application requirements, including real-time constraints and available resources. The preliminary experimental results indicate that the proposed GPSCRS can effectively detect changes in stability under real-time conditions, highlighting its potential for use in remote health monitoring, fall prevention, and rehabilitation scenarios. By providing a quantitative measure of stability, the system enables early risk identification and supports tailored interventions for improved mobility and quality of life.

## 1. Introduction

Maintaining physical stability is crucial for independent living, particularly among the elderly and individuals with mobility impairments. Falls are a significant public health concern, leading to injuries, hospitalizations, and decreased quality of life [1]. Existing methods for assessing physical stability often require specialized equipment and clinical settings [2], limiting their applicability for continuous, at-home monitoring. This paper introduces a gesture-based system using an Arduino microcontroller and the DFRobot Gesture and Touch sensor. This system provides a simple, portable, and cost-effective solution for assessing physical stability based on gesture analysis. By analyzing the timing and consistency of “up” and “down” hand gestures, the system computes a Physical Stability Index (PSI), offering a quantitative measure of an individual’s hand movement control. The system’s focus on capturing temporal changes in gestures, rather than complex movements, is an important design consideration for practicality in real-world applications.

## 2. Literature Review

Wearable sensor technology has emerged as a promising approach for continuous physical stability assessment and fall detection. Accelerometers and gyroscopes are commonly used to measure body movements and detect falls [3,4], but those systems can be susceptible to false alarms due to activities of daily living.

Table 1 presents a comprehensive overview of human physical stability classification methods.

### 2.1. Key Considerations for Method Selection

#### 2.1.1. Sensor Placement Considerations

- Waist/Center of Mass: most common for overall stability assessment.
- Multiple Body Segments: enhanced detail but increased complexity.
- Chest/Sternum: good for respiratory and postural coupling.

#### 2.1.2. Application-Specific Recommendations

- Clinical Assessment: force platforms remain gold-standard.
- Field Studies: IMU-based systems preferred for portability.
- Continuous Monitoring: wearable accelerometers most practical.
- Research Applications: machine learning approaches for pattern discovery.

#### 2.1.3. Performance Metrics

- Accuracy: force platforms > IMU systems > observational methods.
- Portability: wearable sensors > clinical equipment.
- Cost-Effectiveness: IMU systems > force platforms > vision systems.
- Real-time Capability: accelerometers > force platforms > complex ML systems.

Gesture recognition offers an alternative approach by analyzing the patterns and characteristics of movements. Wearable gesture sensors, particularly those using infrared (IR) technology, provide a more portable and versatile solution for activity recognition and fall detection [45].

Based on the comprehensive literature review presented, our proposed GPSCRS offers several distinct advantages over existing methods. Unlike expensive force platforms that require specialized laboratory settings or complex multi-sensor IMU systems that demand sophisticated data processing, our approach provides a cost-effective, portable solution using readily available Arduino microcontrollers and DFRobot Gesture and Touch sensors [46]. The system’s focus on simple “up” and “down” hand gestures eliminates the complexity of multi-dimensional movement analysis while maintaining sensitivity to temporal stability patterns. This simplicity translates to reduced computational requirements, making real-time monitoring feasible. Additionally, our non-invasive gesture-based approach addresses privacy concerns associated with computer vision systems while avoiding the subjective assessment limitations of clinical observational methods like the Berg Balance Scale. The system generates a single Physical Stability Index (PSI), providing clinicians and caregivers with an intuitive, quantitative measure. This index bridges the gap between laboratory-grade precision and practical home-based monitoring, making it particularly valuable for continuous fall prevention and rehabilitation applications in elderly populations.

The DFRobot Gesture and Touch sensor, based on the APDS-9960 chip, is a compact and versatile device that combines gesture recognition and proximity sensing capabilities using infrared (IR) technology. It has been used in various applications, including human–computer interactions and robotics. However, its application in the physical stability assessment is relatively unexplored.

Arduino microcontrollers have been widely adopted in wearable sensor systems due to their low cost, ease of programming, and extensive community support [47]. They provide a flexible platform for data acquisition, processing, and communication.

This work builds upon the existing literature by presenting a novel approach to a physical stability assessment using the DFRobot Gesture and Touch sensor and an Arduino microcontroller. The system focuses on analyzing the timing and consistency of simple hand gestures to quantify movement control and compute a Physical Stability Index (PSI).

For physical stability classification, this study implemented several deep learning models, including Transformer [48,49], Convolutional Neural Networks (CNN) [50,51], and Kolmogorov–Arnold Networks (KAN) [52], in addition to the XGBoost [53] machine learning model. Classification is based on three features extracted from the up–down gesture sequence: the smoothness score, consistency score, and speed score.

## 3. Methodology

### 3.1. System Architecture

The system consists of the following hardware and software components:

- Arduino Uno Microcontroller: serves as the central processing unit for data acquisition, processing, and control.
- DFRobot Gesture and Touch Sensor: Detects “up” and “down” hand gestures. This sensor integrates the APDS-9960 gesture and proximity sensor.
- Serial Communication: used for communication with a computer for data logging and visualization.
- Power Supply: provides power to the Arduino and sensor via USB.

**Figure 1:**
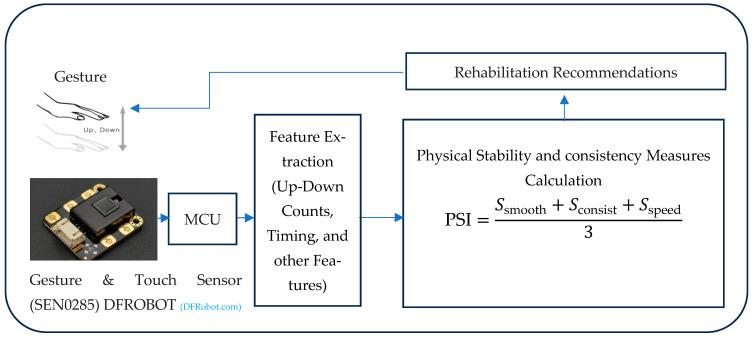
System block diagram.

**Figure 2:**
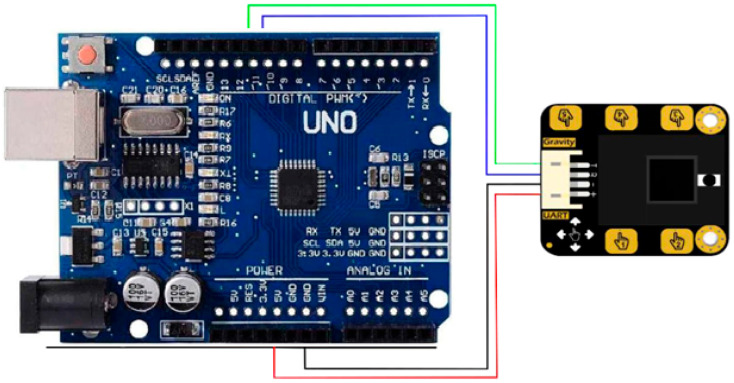
Connection diagram for MCU and gesture sensor interface.

The DFRobot Gesture and Touch sensor [46] is connected to the Arduino Microcontroller Unit (MCU) using I2C communication. The sensor is positioned to capture the user’s hand gestures. The Arduino continuously reads data from the sensor, processes it, and calculates the Physical Stability Index (PSI). Figure 1 shows the System Block Diagram. Figure 2 shows the connection diagram for MCU and gesture sensor interface.

This sensor module integrates gesture recognition and touch detection functions on a breakout board and provides an adjustable detection range within 0–30 cm. When connected to the microcontroller, it can detect a 5-way touch signal and seven types of gestures: move left, move right, move forward, move backward, pull up, pull down, pull, and remove. The sensor is also equipped with an auto-sleep and wake-up function. The module features an integrated gesture recognition algorithm and provides a simple and reliable data output. The sensor communicates directly with the Arduino microcontroller via a serial port. The maximum height at which the sensor can detect a user’s hand movements is 30 cm.

**Figure 3:**
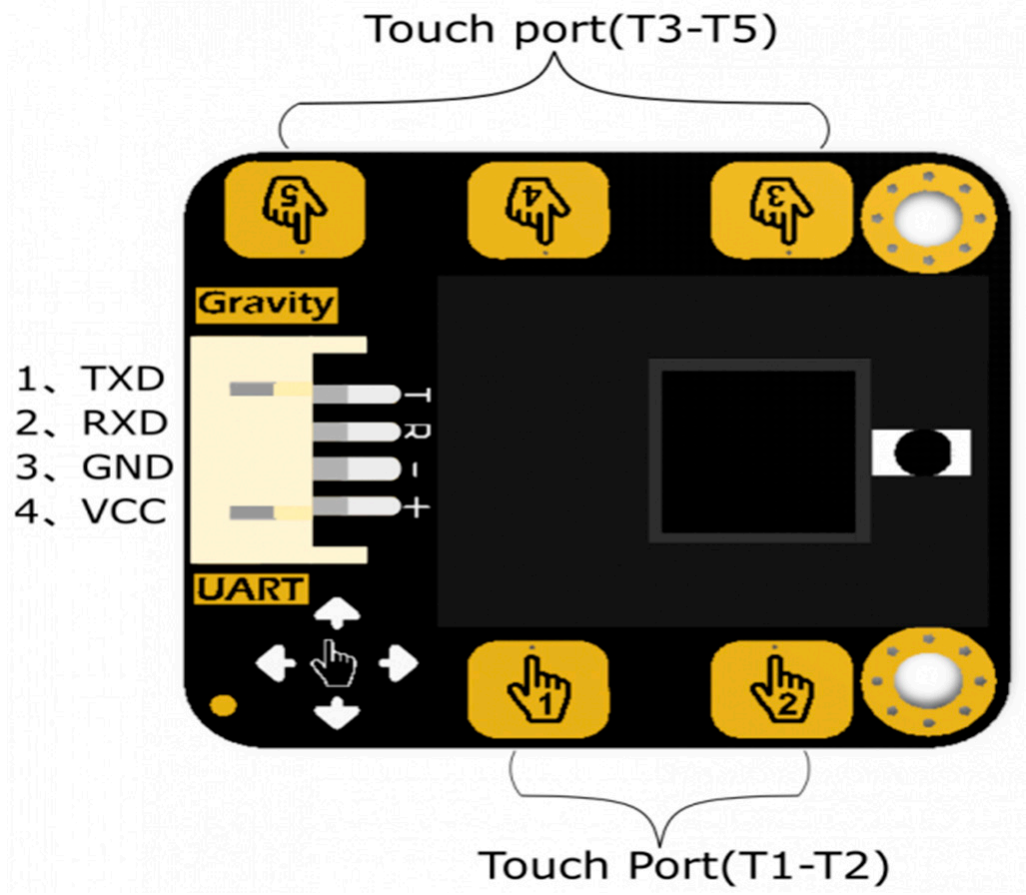
Touch sensor ports (www.DFRobot.com).

**Figure 4:**
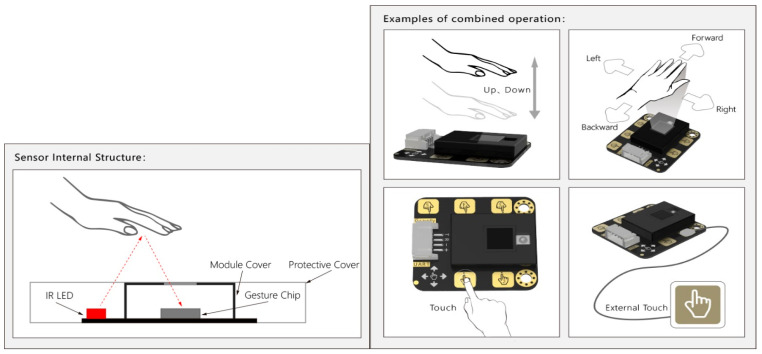
Principle of work of the gesture detection sensor using an IR LED (www.DFRobot.com).

Figure 3 shows the ports of the touch sensor. The outer shield for the sensor retains the advantages of the gravity series as well as making the sensor more durable. Figure 4 shows the principle of gesture detection using an IR LED. The maximum height the sensor can detect is 30 cm.

### 3.2. Feature Extraction

**Figure 5:**
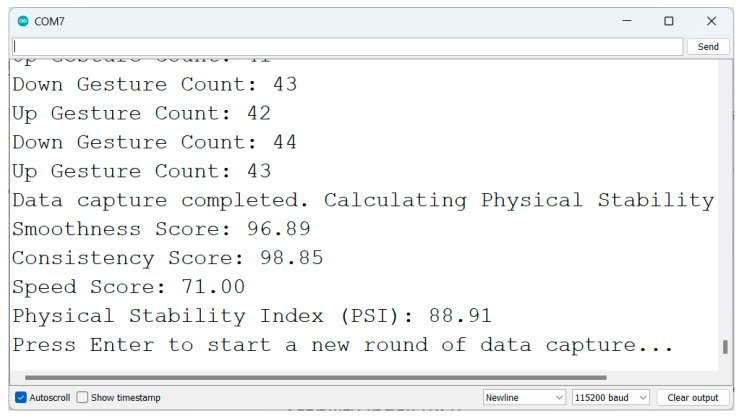
Gesture detection, feature extraction, and PSI calculation results.

Figure 5 shows the results of gesture detection, feature extraction, and the calculation of the Physical Stability Index (PSI).

#### 3.2.1. Gesture Counting and Timing Feature Extraction

The system counts and timestamps “Up” and “Down” gestures. The timestamps are stored in circular buffers for analysis.

- Let $t_{current}$ be the current time in milliseconds.
- Let $t_{last}$ be the timestamp of the last gesture of the same type.
- Let $\Delta t_{debounce}=200\;ms$ be the debounce time to filter out consecutive gestures.

A gesture is counted if:

(1) $$t_{current\;}-t_{last\;}>\Delta t_{debounce}$$

The timestamps for “Up” and “Down” gestures are stored in arrays $T_{up\;}$ and $T_{down,\;}$, respectively, with a circular buffer of size 10.

Three scores are calculated to classify the human physical stability: the Smoothness Score, Consistency Score, and Speed Score.

The relevance of smoothness to physical stability classification has been demonstrated in [54]. It was established in [55] that movement smoothness is a valid indicator of motor control and neurological function. Regarding consistency, research in [56] demonstrated that movement variability and consistency are clinically relevant markers of stability and fall risk. For speed-related metrics, the extensive literature on the Timed Up and Go (TUG) test, including seminal work in [29,30], has established the movement speed as a validated predictor of a fall risk and functional mobility.

#### 3.2.2. Smoothness Score Calculation

The smoothness score measures the deviation in timing between consecutive pairs of “Up” and “Down” gestures.

- Let $T_{up}=\left[t_{up,1},t_{up,2},\ldots ,t_{up,10}\right]$ be the timestamps for “Up” gestures.
- Let $T_{down\;}=\left[t_{down,1},t_{down,2},\ldots ,t_{down,10}\right]$ be the timestamps for “Down” gestures.
- Let $\Delta t_{up,i}=t_{up,i}-t_{up,i-1}$ be the interval between consecutive “Up” gestures.
- Let $\Delta t_{down,i}=t_{down,i}-t_{down,i-1}$ be the interval between consecutive “Down” gestures.

The deviation $d_{i}$ for each pair of intervals is

(2) $$d_{i}=\frac{\left|\Delta t_{up,i}-\Delta t_{down,i}\right|}{\left(\Delta t_{up,i}+\Delta t_{down,i}\right)/2}$$

The total deviation D is the average of all valid deviations:

(3) $$D=\frac{1}{N}\sum_{i=1}^{N}d_{i}$$

where N is the number of valid pairs.

The smoothness score $S_{smooth}$ is

$$S_{smooth}=100-\left(D\times 100\right)$$

(4) $$S_{smooth}=100-\left(\frac{1}{N}\sum_{i=1}^{N}\frac{\left|\Delta t_{up,i}-\Delta t_{down,i}\right|}{\left(\Delta t_{up,i}+\Delta t_{down,i}\right)/2}\times 100\right)$$

#### 3.2.3. Consistency Score Calculation

The consistency score measures the balance between “Up” and “Down” gestures.

- Let $N_{up}$ be the count of “Up” gestures.
- Let $N_{down}$ be the count of “Down” gestures.
- Let $N_{total}=N_{up}+N_{down}$ be the total number of gestures.

The ratios of “Up” and “Down” gestures are

(5) $$r_{up}=\frac{N_{up}}{N_{total}},r_{down}=\frac{N_{down}}{N_{total}}$$

The maximum deviation Δr is

(6) $$\Delta r=\left|r_{up}-r_{down}\right|$$

The consistency score $S_{consist}$ is

(7) $$S_{consist}=100-(\Delta r\times 100)$$

#### 3.2.4. Speed Score

The speed score evaluates how quickly the fastest gesture was performed. It is calculated as follows:

a Normalization:

The fastest gesture time (Fastest Gesture Time) is mapped to a score between 0 and 100 using the formula:

Speed Score = map(Fastest Gesture Time, 50, 1000, 100, 0)(8)

where:

- 50 ms represents the fastest possible gesture time.
- 1000 ms represents the slowest acceptable gesture time.

The mapping function can be expressed mathematically as:

Speed Score = 100 − (1000 − 50 Fastest Gesture Time − 50 × 100)

b Constraints:

The speed score is constrained to lie within the range [0, 100]:

Speed Score = max(0, min(100, Speed Score))

#### 3.2.5. Physical Stability Index (PSI)

The PSI is an average of the smoothness, consistency, and speed scores.

- Let $S_{smooth}$ be the smoothness score.
- Let $S_{consist}$ be the consistency score.
- Let $S_{speed}$ be the speed score.

The PSI is calculated as:

(9) $$PSI=\frac{S_{smooth}+S_{consist}+S_{speed}}{3}$$

#### 3.2.6. Data Capture Duration

The system captures data for a fixed duration of 40 s.

- Let $t_{start}$ be the start time of the data capture.
- Let $t_{current}$ be the current time.
- Let $\Delta t_{capture}=40,000\;ms$ be the data capture duration.

The data capture stops when:

(10) $$t_{current}-t_{start}\geq \Delta t_{capture}$$

### 3.3. Physical Stability Index Calculation Algorithm

The algorithm for the physical stability assessment involves the following steps.

#### 3.3.1. Initialization

- Initialize the serial communication for debugging and data logging.
- Configure the DFRobot Gesture and Touch sensor for “up” and “down” gesture detection. This includes setting the gesture distance and enabling the appropriate functions.

#### 3.3.2. Data Acquisition

- Continuously monitor the DFRobot Gesture and Touch sensor for gesture events. The DFGT.getAnEvent() function is used to retrieve the detected gesture.
- Record the timestamps of “up” and “down” gestures using the millis() function.

#### 3.3.3. Gesture Processing

- Debounce the gestures to prevent multiple counts for a single gesture. This is achieved by ensuring a minimum time interval (debounceTime = 200 ms) between consecutive gestures.
- Store the timestamps of the last 10 “up” and “down” gestures in circular buffers (upTimes[] and downTimes[]).

#### 3.3.4. Physical Stability Calculation

- Smoothness Score: Calculate the smoothness score based on the consistency of time intervals between consecutive gestures. The calculated Smoothness() function computes the average deviation between the intervals of “up” and “down” gestures. A lower deviation indicates smoother movements and a higher smoothness score.
- Consistency Score: Calculate the consistency score based on the ratio of “up” and “down” gestures. The calculated Consistency() function penalizes large deviations from an equal distribution of “up” and “down” gestures. The greater the evenness of the gestures, the higher the score.
- Speed Score: it represents the speed of the gestures.
- Overall PSI: Combine the smoothness, consistency, and speed scores to calculate the overall Physical Stability Index (PSI). The PSI is computed as the average of the three scores.

#### 3.3.5. Data Output

- Print the smoothness score, consistency score, speed score, and PSI to the serial monitor.

#### 3.3.6. Looping and Resetting

The system captures data for a fixed duration (dataCaptureDuration = 40,000 ms). After the data capture is complete, the PSI is calculated and displayed. The system then waits for the user input to start a new round of data capture. All variables are reset using the function resetSystem() for the new round.

### 3.4. Models Architectures and Their Computational Complexities

Table 2 summarizes the implemented model architectures and their computational complexities.

The computational complexity analysis reveals distinct trade-offs between the training efficiency and model sophistication across the four evaluated architectures. The CNN and transformer models exhibit traditional iterative training complexities that scale with the number of samples and architectural parameters, with the transformer’s simplified single-sequence implementation reducing the typical quadratic attention complexity. KAN presents a unique one-shot training approach with polynomial complexity in expanded features, offering extremely fast predictions once trained. XGBoost demonstrates the most balanced profile, leveraging histogram-based optimization for efficient training that scales well with large datasets while maintaining very fast tree-traversal predictions. The choice between these models should consider not only accuracy requirements, but also the computational budget available for both training and inference phases of deployment.

### 3.5. Classifier Model Architectures

The architectures of the four implemented models are detailed below, outlining the essential components, layers, and configuration for each.

#### 3.5.1. Transformer Neural Network Model Architecture

The transformer model [48] leverages attention mechanisms for sequence classification tasks. The framework initiates with an Input Embedding layer that converts input features into a higher-dimensional embedding space, followed by Positional Encoding to incorporate positional information within the input sequence representing one feature value set.

The transformer encoder’s core lies within the Encoder Layer, which implements multi-head self-attention through the Multi Head Attention layer, enabling the model to assess feature significance within the input. A feed-forward network subsequently processes the transformed representation. Layer normalization is applied to both attention and feed-forward outputs to enhance training stability and maintain consistent output ranges.

The output undergoes global average pooling before reaching the classification layers. The Transformer Classifier class encompasses all layers and defines forward propagation through the call method. The model incorporates a custom train_step method for training and a get_config method for model saving and loading.

The model is compiled using the ADAM optimizer with categorical cross-entropy loss and accuracy metrics, then trained to predict labels and generate classification reports, ROC curves, and confusion matrices.

#### 3.5.2. Convolutional Neural Network (CNN) Architecture

The CNN [51] processes sequential gesture-based data through convolutional operations to extract meaningful features. Input features are reshaped to dimensions (3, 1) for 1D convolutional operations.

The network’s core consists of two 1D convolutional layers (Conv1D): the first uses 64 filters with kernel size 3, while the second employs 32 filters with an identical kernel size. These layers perform convolutions on the reshaped input, generating feature maps that emphasize relevant patterns within gesture stability data.

A Flatten layer converts feature maps into a 1D feature vector for fully connected processing. The flattened output passes through a dense layer before reaching the final classification layer. The CNN’s key parameters include filter configurations of (64, 32), a kernel size of 3, enabling classification through temporal and spatial analysis of gesture-based movement patterns.

#### 3.5.3. XGBoost (Extreme Gradient Boosting) Architecture

- XGBoost (Extreme Gradient Boosting) [57] is a highly optimized and extensively utilized implementation of the gradient boosting framework, specifically engineered for the speed, efficiency, and predictive performance. Similar to standard Gradient Boosting, it employs an ensemble learning approach that classifies by sequentially training decision trees in a stage-wise manner.
- This model accepts the three scaled input features (the Smoothness Score, Consistency Score, and Speed Score) derived from gesture-based stability assessments and builds an ensemble of decision trees. Trees are added iteratively, with each successive tree trained to predict and minimize residual errors (using gradient information) remaining from the previous ensemble. XGBoost’s key distinguishing features include integrated L1 and L2 regularization on leaf weights to prevent overfitting and various system optimizations for accelerated training.
- The model processes the gesture-derived features through multiple boosting rounds, where each decision tree learns to classify the four stability categories: Stable, Highly Stable, Unstable, and Highly Unstable. The ensemble approach is particularly effective for this application as it can capture complex non-linear relationships between the three input features and their corresponding stability classifications.
- Key parameters used for this XGBoost model include 100 boosting stages (n_estimators), a learning rate of 0.1, a maximum depth of 6 for individual trees (max_depth), a random state of 42, and it optimizes a multi-class logarithmic loss (‘mlogloss’) function suitable for the four-class stability classification problem.

#### 3.5.4. Kolmogorov–Arnold Network (KAN) Architecture

The Kolmogorov–Arnold Network (KAN) [52,58] is a classification methodology inspired by the Kolmogorov–Arnold representation theorem, adapted here for classification purposes. Unlike iterative approaches such as boosting or deep neural networks, this model operates in a single stage by initially transforming scaled input features using a fixed set of non-linear basis functions.

Subsequently, a single linear layer maps these expanded features directly to class the prediction scores. The model’s parameters (the weights of this final linear layer) are determined efficiently in one step through a direct pseudoinverse calculation based on the expanded features and target labels, rather than through iterative gradient-based optimization. Key defining characteristics for this specific implementation include the degree 3 polynomial feature expansion and the direct, non-iterative training methodology.

## 4. Results and Discussion of Physical Stability Calculation

**Figure 6:**
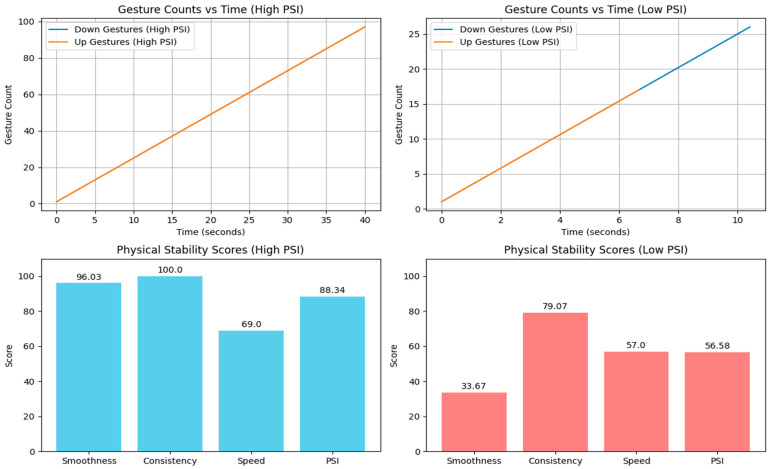
Up and down gesture counts with their corresponding Physical Stability Scores.

Figure 6 shows gesture counts over time for two sample test cases: one with a high PSI and the other with a low PSI and their corresponding Physical Stability Scores.

The system was tested on ten healthy volunteers and the mean PSI score was 87.5 with a standard deviation (SD) of 5.2. Volunteers were then asked to perform the gesture task while simulating slight instability (e.g., pretending to be dizzy). In these conditions, the mean PSI dropped to 75.3 (SD = 7.8), indicating a decrease in stability. Table 3 shows the feature values and their corresponding PSI for two cases.

## 5. Physical Stability Classification Using Both Deep Learning and Machine Learning

The goal is to classify gestures into four stability categories: Stable, Highly Stable, Unstable, and Highly Unstable. The input consists of features such as smoothness, consistency, speed, and PSI (Equation (10)) which are extracted from the user-generated sequence of Up and Down hand gestures. The output is the predicted stability class for each gesture.

### 5.1. Gesture-Based Physical Stability Classification

#### 5.1.1. Data Generation

Gesture data are acquired from the sensor and used to calculate the features for each stability category:

- Stable: moderately high values for all features.
- Highly Stable: high values for smoothness, consistency, speed, and PSI.
- Unstable: moderate values for some features and lower values for others.
- Highly Unstable: low values for most features.

Table 4 shows the physical conditions of the participants.

#### 5.1.2. Data Preprocessing

Before feeding the data into models, the following preprocessing steps are applied:

1. Standardization: features are scaled using Standard Scalar to ensure consistent scaling across all features.
2. Data Splitting: the dataset is divided into training and testing sets (or cross-validation folds) to evaluate the model performance.

#### 5.1.3. Model Selection

Multiple models are defined for classification, including both traditional machine learning and deep learning approaches:

- Deep Learning Models:

1. Convolutional Neural Network (CNN): captures spatial patterns in the data.
2. Transformer-based Classifier: handles sequential data effectively.
3. Kolmogorov–Arnold Network (KAN): The Kolmogorov–Arnold representation theorem, states that any continuous multivariate function can be represented as a superposition of univariate functions and additions. KANs are designed to directly learn this representation from data.

- Traditional Machine Learning Models:

XGBoost: a gradient-boosting framework.

#### 5.1.4. Model Training

For each model:

- Training and Validation Split: the training data are further divided into training and validation subsets with K-Fold Cross-Validation.
- Optimization: models are trained using appropriate optimization techniques (e.g., Adam optimizer, Cross-Entropy Loss).
- Early Stopping: training is stopped early if validation loss does not improve for a certain number of epochs to prevent overfitting.

#### 5.1.5. Model Evaluation

After training, each model is evaluated on the test set using the following metrics:

- Accuracy: proportion of correctly classified samples.
- Precision, Recall, and F1-Score: metrics for evaluating the balance between false positives and false negatives.
- Confusion Matrix: a visual representation of the classification performance across classes.
- ROC Curves: plots of true-positive rate vs. false-positive rate for each class.

#### 5.1.6. Cross-Validation

To ensure robust evaluation, K-Fold Stratified Cross-Validation is performed:

1. The dataset is split into K folds.
2. Each fold is used once as a test set while the remaining folds are used for training.
3. Performance metrics are aggregated across all folds for each model.

Table 5 summarizes the comparative performance of all models:

- Mean Accuracy: average accuracy across all folds.
- Precision, Recall, and F1-Score: weighted averages of these metrics across all folds.
- The best-performing model is identified based on these metrics.

The proposed algorithm provides a comprehensive framework for gesture-based physical stability classification. By leveraging both traditional machine learning and deep learning models, the system ensures robustness and flexibility in handling diverse datasets. The systematic evaluation of each model allows for the selection of the most effective model for deployment in real-world applications.

The performance metrics for the models were evaluated across five folds using Stratified K-Fold Cross-Validation. The key metrics considered were Recall, Mean Accuracy, Precision, and F1-Score. Below is a detailed analysis of the results:

#### 5.1.7. Results of Deep Learning Models

- Transformer:

1. Achieved perfect scores (99.50%) across all metrics.
2. This indicates that the transformer model excelled at capturing complex patterns in the data, likely due to its attention mechanism, which allows it to focus on relevant features effectively.
3. However, its computational complexity is high due to quadratic scaling with the sequence length, making it less suitable for real-time or resource-constrained applications.

- CNN:

1. Performed very well with an accuracy of 99.00% and an F1-Score of 98.99%.
2. CNNs are effective at extracting spatial features, which may explain their strong performance on this dataset.
3. The model’s computational cost is moderate compared to transformers, but still higher than traditional machine learning methods.

- KAN:

1. Resulted in an accuracy of 88.8% and an F1-Score of 86.63%.

#### 5.1.8. Results of Traditional Machine Learning Models

- XGBoost:

1. Performed well, with an accuracy of 98.00% and an F1-Score of 97.99%.
2. XGBoost is known for its efficiency and ability to handle imbalanced datasets, which may explain its slightly lower performance compared to DL models.

Deep learning models (transformer, CNN, and KAN) offer a superior performance but come at the cost of higher computational complexity. Traditional machine learning models (XGBoost) provide a good balance between the performance and computational efficiency.

## 6. Dataset Characteristics

**Figure 7:**
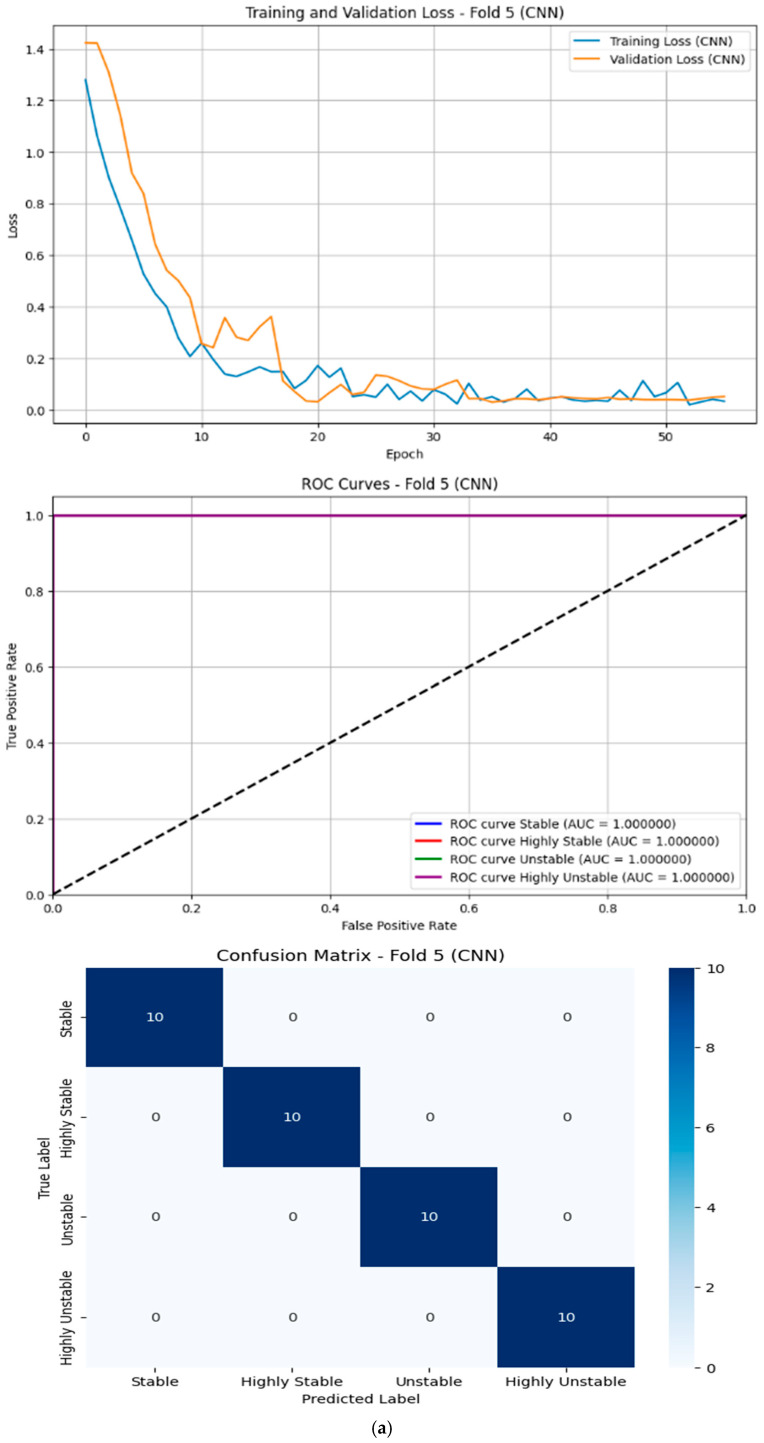

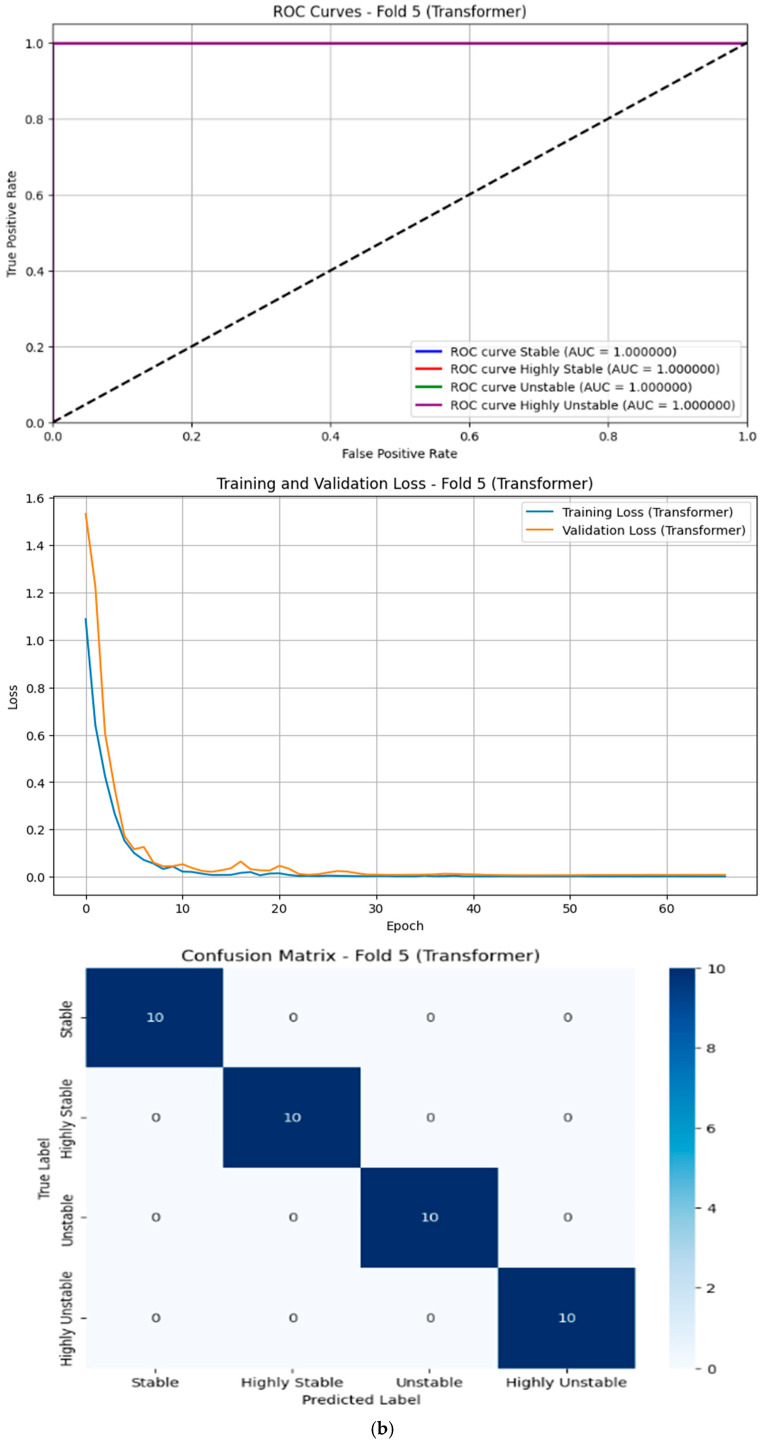

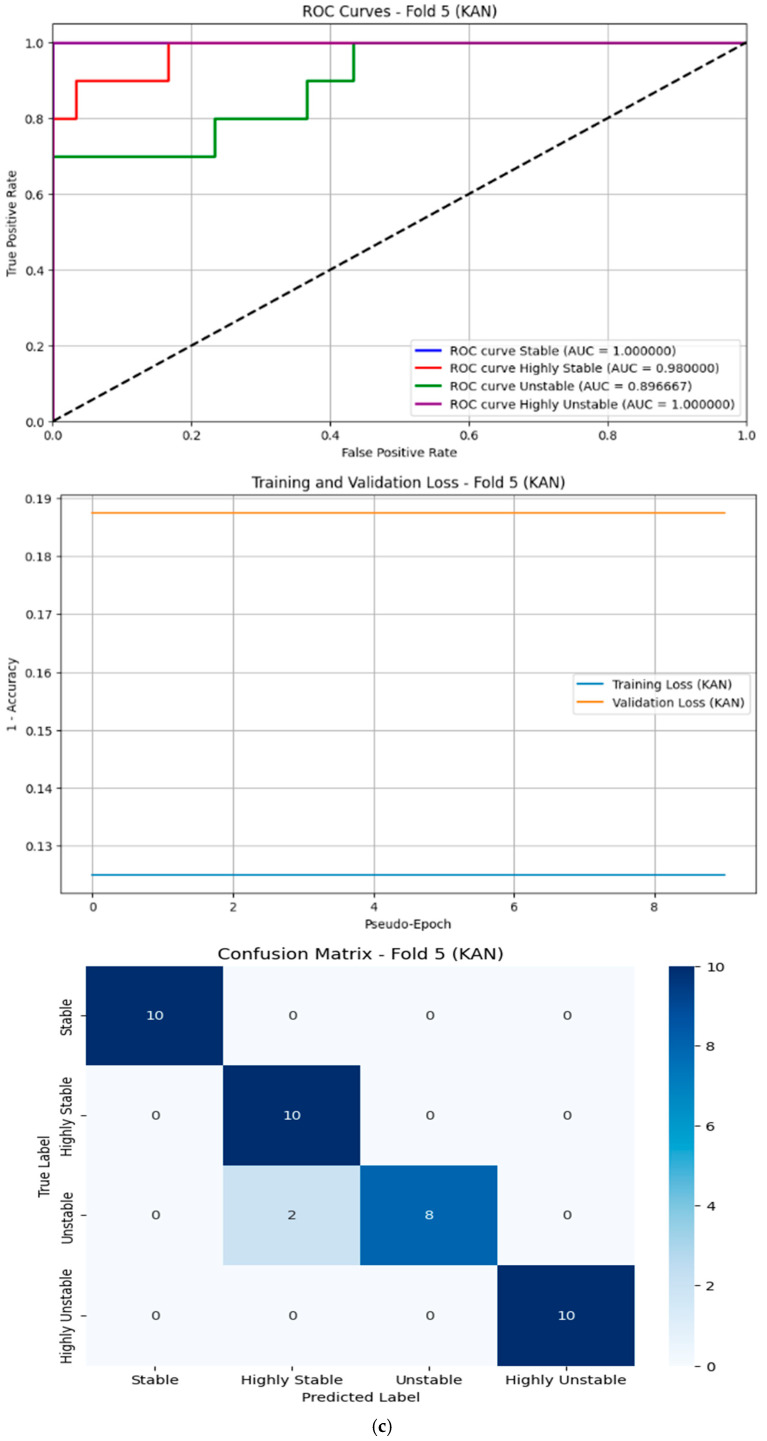

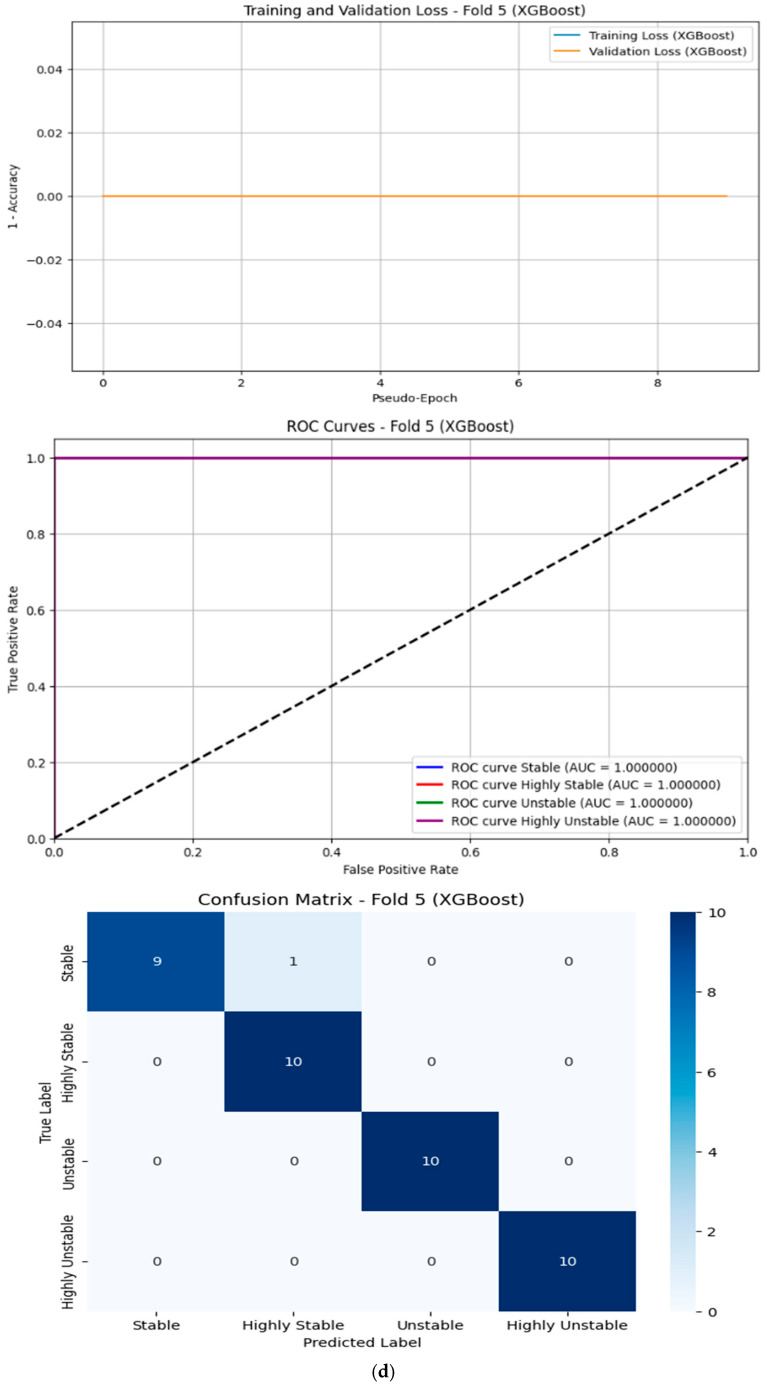
ROC, Loss Curves, and Confusion Matrices (Fold 5) for different models. (**a**) CNN Model. (**b**) Transformer Model. (**c**) KAN Model. (**d**) XGBoost Model.

The dataset appears to have clear separability between classes, as evidenced by the high performance of most models. This study demonstrates the effectiveness of various models for gesture-based physical stability classification. While deep learning models excel in terms of their performance, traditional machine learning models like XGBoost offer a compelling alternative with lower computational requirements. Future work could explore hybrid approaches or ensemble methods to further enhance the performance while maintaining efficiency. Figure 7 shows the ROC, Loss Curves, and Confusion Matrices for the following models: (a) CNN, (b) Transformer, (c) KAN, and (d) XGBoost.

## 7. Gesture-Based Stability Classification for Rehabilitation

The Gesture-Based Stability Classification, derived from the analysis of hand gestures, represents a novel approach to enhancing rehabilitation strategies. By leveraging gesture sensor technology, this system offers a personalized, engaging, and cost-effective solution for assessing and improving the physical stability. The system classifies users into four distinct categories—Stable, Highly Stable, Unstable, and Highly Unstable—based on three score (the Smoothness Score, Consistency Score, and Speed Score), enabling targeted interventions. This classification enhances the precision of rehabilitation programs across various contexts, including stroke recovery, elderly fall prevention, and sports injury rehabilitation.

### 7.1. Establishing a Baseline Stability

Before initiating a rehabilitation program, it is essential to establish a baseline GBSC. Users perform standardized hand gestures (e.g., Up and Down) for a set duration (e.g., 40 s). The system records initial scores for the smoothness, consistency, and speed, which collectively determine the user’s stability classification: Stable, Highly Stable, Unstable, or Highly Unstable. For example, an initial score of 75.01 with a smoothness score of 54.05 and a consistency score of 70.97 might classify the user as “Unstable”. This classification serves as a reference point for tracking progress throughout the rehabilitation process.

### 7.2. Designing Personalized Exercise Programs

The system and its associated classification inform the creation of individualized exercise regimens tailored to address specific weaknesses identified by the system. Low smoothness scores suggest uncoordinated movements requiring motor control exercises, while low consistency scores indicate imbalanced gesture execution necessitating balanced muscle engagement exercises. Similarly, low speed scores require stretching/flexibility and strength-building exercises. Example exercises include the following:

- Smoothness Improvement: slow, controlled hand gestures executed with minimal deviation.
- Consistency Improvement: rhythmic alternation between Up and Down gestures.
- Speed Improvement: gradual increase in the pace of gesture execution over time.

For users classified as “Highly Unstable,” the program may initially focus on foundational exercises to improve the overall stability before progressing to more advanced activities.

### 7.3. Tracking Progress over Time

Regular monitoring facilitates adaptive rehabilitation strategies. Periodic data capture (e.g., weekly or bi-weekly) allows comparison of updated scores with previous results to assess progress. Changes in stability classification guide adjustments to the program:

- Improvement: a shift from “Unstable” to “Stable” or “Highly Stable” indicates significant progress, warranting increased exercise difficulty.
- Plateau or Decline: persistent “Unstable” or “Highly Unstable” classifications necessitate re-evaluation and modification of the program.

For instance, progressing from a score of 75.01 in week 1 to 87.15 in week 2, coupled with improvements in smoothness and consistency, might elevate the user’s classification from “Unstable” to “Stable,” prompting the introduction of more challenging exercises.

### 7.4. Providing Real-Time Feedback

Real-time feedback enhances user engagement and accelerates learning. During sessions, the system displays metrics such as gesture counts and highlights deviations from expected patterns, prompting corrective actions. Verbal or visual cues like “Move more smoothly” or “Balance your gestures” guide users toward the proper technique. For example, if movements become jerky, the system advises focusing on smoother transitions, helping users maintain or improve their stability classification.

### 7.5. Gamifying the Rehabilitation Process

Gamification transforms rehabilitation into an enjoyable experience. Elements such as score improvement targets, awarding points/badges for milestones, and progressively challenging exercises motivate users. Interactive visuals or auditory feedback further enhance engagement. An example challenge might involve performing 40 gestures with a smoothness score above 70, rewarding users upon successful completion. Achieving these goals can help users transition from “Unstable” to “Stable” or even “Highly Stable.”

### 7.6. Collaborating with Healthcare Providers

Seamless integration with healthcare professionals ensures informed rehabilitation. Sharing GBSC data and stability classifications enables therapists to remotely review scores and suggest adjustments. This collaboration ensures adherence to professional recommendations and leverages expert insights. For instance, a physical therapist reviewing a user’s GBSC history might recommend incorporating strength exercises to improve the speed score further, helping the user progress toward a “Highly Stable” classification.

The following rehabilitation professionals (Table 6) are typically qualified to apply the Physical Stability Classification System (Stable, Highly Stable, Unstable, Highly Unstable), which uses up-and-down hand gestures, for therapeutic purposes:

As those experts already employ gesture-recognition systems (EMG, IMU, or vision-based) for upper-extremity rehabilitation, they are well positioned to adopt a simple hand-gesture stability index as an additional clinical decision-support tool.

### 7.7. Long-Term Monitoring and Maintenance

Sustaining gains and preventing regression are critical for long-term success. Periodic assessments continue even after achieving rehabilitation goals, monitoring the GBSC for early signs of decline. Consistent adherence to maintenance exercises ensures sustained stability. For example, users might conduct monthly assessments post-rehabilitation to ensure they remain in the “Stable” or “Highly Stable” category.

### 7.8. Enhancing Functionality with Additional Sensors

Incorporating additional sensors, such as accelerometers and gyroscopes, augments the system’s capabilities. These devices refine measurements of range of motion and speed, enabling more comprehensive assessments. Machine learning algorithms can be used to predict recovery trends and personalize programs further. For instance, adding an accelerometer improves the range of motion accuracy by precisely measuring the gesture amplitude, aiding users in achieving and maintaining a “Highly Stable” classification.

#### Benefits of Gesture-Based Stability Classification for Rehabilitation

The integration of a gesture-based stability classification system into rehabilitation programs offers several advantages:

1. Personalization: tailored exercises address individual needs and weaknesses based on objective data, including stability classification.
2. Engagement: gamification and real-time feedback maintain motivation and adherence, helping users achieve higher stability classifications.
3. Accuracy: smoothness, consistency, and speed-based classifications provide quantitative measures of progress.
4. Flexibility: the system supports remote use and complements in-person therapy, expanding accessibility.
5. Cost-Effectiveness: reduced need for frequent in-person visits lowers costs while maintaining high-quality care.

By empowering users to actively manage their health and track their stability classification, the system promotes a proactive and holistic approach to rehabilitation. It holds promise for diverse applications, from stroke recovery to elderly care and sports rehabilitation. Future research and development, particularly focusing on incorporating additional sensors such as accelerometers and refining algorithms, will unlock its full potential.

## 8. Conclusions and Future Work

This paper introduced a novel approach for the Gesture-Based Physical Stability Classification and Rehabilitation System (GPSCRS) and evaluated the effectiveness of various machine learning and deep learning models for gesture-based physical stability classification. This study utilized a low-cost, portable system built around an Arduino microcontroller and the DFRobot Gesture and Touch sensor. The system computes a Physical Stability Index (PSI) by analyzing “up” and “down” hand gestures. Among the models tested, neural network architectures such as Transformers, KAN, and CNN demonstrated near-perfect performance metrics (Mean Accuracy, Recall, Precision, and F1-Score of 1.0), while traditional machine learning models like XGBoost also showed strong results, offering a trade-off between the accuracy and computational efficiency.

The choice of a model should align with specific application requirements, considering factors such as accuracy needs, computational resources, and real-time constraints. Traditional models like XGBoost provide compelling alternatives with lower resource demands, making them suitable for embedded systems or environments with limited processing power.

The preliminary results indicate that the system can effectively detect changes in stability under real-time test conditions. Future work will focus on:

1. Expanding Stability Assessment Parameters: adding accelerometer measurements to analyze the speed, range of motion, and tremor patterns for a more comprehensive stability evaluation.
2. Validation on Diverse Populations: testing the system on larger and more varied groups, including elderly individuals and those with mobility impairments, to ensure its applicability across different demographics.
3. Cloud Integration: developing a cloud-based platform for remote monitoring and data analysis to facilitate broader usage and accessibility.
4. Real-Time Feedback Mechanisms: implementing feedback systems to assist users in improving their stability and preventing falls.

The proposed system offers a cost-effective and straightforward approach to assessing physical stability, providing a quantitative measure of stability that can help identify risks and guide interventions. By integrating additional sensors, future iterations of the system could further enhance its capabilities, ultimately contributing to physical stability and an improved quality of life for at-risk populations.

## Figures and Tables

**Table 1:** Human physical stability classification methods—summary.

Method	Sensors	Key References	Pros	Cons
Force Platform-Based Methods				
Center of Pressure (COP) Analysis	Force plates, pressure sensors	[5,6,7]	Gold standard for static balance High precision and accuracy Well-established normative data Comprehensive biomechanical analysis	Expensive equipment Limited to laboratory settings Cannot assess dynamic activities Requires specialized facilities
Static Posturography	Multi-axis force platforms	[8,9]	Standardized testing protocols Quantitative measurements Sensitive to balance deficits	Limited ecological validity Static conditions only High cost and space requirements
Inertial Measurement Unit (IMU)-Based Methods				
Accelerometer-Based Stability	Tri-axial accelerometers	[10,11,12]	Portable and wearable Low cost Real-time monitoring Suitable for field studies	Less accurate than force platforms Sensitive to sensor placement Limited frequency response
Multi-Sensor IMU Systems	Accelerometers, gyroscopes, magnetometers	[13,14,15]	Comprehensive motion capture Dynamic balance assessment Multiple body segment tracking	Complex data processing Sensor fusion challenges Battery life limitations
Pendant-Mounted Systems	Single IMU at center of mass	[16,17]	Simple setup Natural wearing position Good for daily monitoring	Limited to trunk movements Reduced sensitivity Single-point measurement
Machine Learning Classification Approaches				
Supervised Learning (SVM, RF, k-NN)	Accelerometers, gyroscopes	[18,19,20]	High classification accuracy Automated analysis Adaptable to different populations	Requires labeled training data Computationally intensive May overfit to training conditions
Unsupervised Learning (k-Means, GMM, HMM)	Inertial sensors	[21,22]	No labeled data required Discovers natural patterns Adaptive to new conditions	Difficult to interpret results Less predictable performance Requires domain expertise
Deep Learning Approaches	Multi-modal sensor data	[23,24,25]	Automatic feature extraction High accuracy potential Handles complex patterns	Requires large datasets Black box nature High computational requirements
Observational and Clinical Methods				
Berg Balance Scale	Visual observation	[26,27,28]	Widely used clinical tool No equipment required Established validity	Subjective assessment Limited sensitivity Ceiling effects
Timed Up and Go (TUG)	Stopwatch, optional IMU	[29,30,31]	Simple and quick Functional assessment Predictive of fall risk	Limited granularity Subjective timing Single-task focus
Multiscale Entropy (MSE) Analysis				
Complexity Analysis	Force plates, accelerometers	[32,33,34]	Captures signal complexity Sensitive to subtle changes Complementary to traditional metrics	Complex interpretation Requires specialized knowledge Sensitive to signal quality
Emerging Technologies				
Computer Vision Systems	RGB cameras, depth sensors	[35,36,37]	Non-contact measurement Rich visual information Multiple subjects simultaneously	Privacy concerns Lighting-dependent Occlusion issues
Hybrid Sensor Systems	IMU + cameras + pressure sensors	[38,39,40]	Comprehensive data capture Improved accuracy Redundant measurements	Increased complexity Higher costs Data fusion challenges
Additional Clinical Assessment Tools				
Tinetti Performance-Oriented Mobility Assessment (POMA)	Visual observation	[41,42]	Comprehensive mobility assessment Standardized scoring Predictive of falls	Time-consuming Requires training Subjective elements
Functional Reach Test	Measuring ruler	[43,44]	Simple to administer Minimal equipment Good inter-rater reliability	One-dimensional assessment Limited dynamic component Ceiling effects

**Table 2:** Model architecture summary.

Model Name	Key Components/Stages
CNN	1. Input reshaped to [batch, 1, features] for 1D processing. 2. Two Convolutional Blocks (Conv1D, BatchNorm, ReLU, MaxPool) for feature extraction. 3. Flattening of feature maps. 4. Fully Connected classification head (Linear, ReLU, Dropout, Linear output).
Transformer	1. Input reshaped to [batch, 1, features] (sequence length 1). 2. Linear projection maps input features to d_model embedding space. 3. Stacked Transformer Encoder layers (Multi-Head Self-Attention, Feed-Forward Network, LayerNorm, Residuals) applied to the single sequence element. 4. Output representation extracted. 5. Classifier head (LayerNorm, MLP, Linear output).
KAN	1. Fixed non-linear feature expansion using polynomial basis functions (up to degree 3). 2. Single linear layer maps expanded features directly to class outputs. 3. Training via one-shot pseudoinverse calculation, not iterative gradient descent.
XGBoost	1. Ensemble of decision trees built sequentially (Gradient Boosting). 2. Each new tree fits the gradient of the loss w.r.t. the previous ensemble’s predictions. 3. Includes L1/L2 regularization on leaf weights. 4. Uses efficient histogram-based algorithm for finding splits.

**Table 3:** Feature values and their corresponding PSI for two sample cases.

Features and Their PSI	Low Stability Case	High Stability Case
Smoothness Score	96.89	94.79
Consistency Score	98.85	99.21
Speed Score	71.00	82.00
Physical Stability Index (PSI)	88.91	92.00

**Table 4:** Physical conditions of participants.

PSI Range	Classification	Physical Condition Indicators
>90	Highly Stable	Optimal Performance Balance and Coordination: smooth, precise movements with minimal deviation from the expected vertical path Muscle Strength and Endurance: consistent force and speed maintained throughout repeated gestures
76–90	Stable	Good Performance with Minor Issues Balance and Co-ordination: generally smooth movements with occasional minor tremors or slight trajectory deviations Muscle Strength and Endurance: adequate strength with minimal fatigue during extended sessions.
60–75	Unstable	Moderate Impairments Balance and Coordination: noticeable tremors, irregular movements, moderate deviations from straight paths Muscle Strength and Endurance: visible decline in movement speed/force over time, muscle fatigue evident Joint Flexibility: limited range of motion, reduced gesture amplitude Neurological Function: delayed responses, moderate asymmetry between dominant/non-dominant arms
<60	Highly Unstable	Significant Impairments Balance and Coordination: severe tremors, highly irregular movements, major trajectory deviations Muscle Strength and Endurance: inability to maintain consistent movements, rapid muscle fatigue, difficulty completing gestures Joint Flexibility: severely restricted range of motion, pain/discomfort during movements Neurological Function: significantly delayed or jerky movements, marked asymmetry, potential neural pathway disruptions

**Table 5:** Performance summary across all models.

Model	Recall	Accuracy	Precision	F1-Score
Transformer	0.9950	0.9950	0.9950	0.9949
CNN	0.9900	0.9903	0.9900	0.9899
KAN	0.8700	0.8880	0.8700	0.8663
XGBoost	0.9800	0.9800	0.9800	0.9799

**Table 6:** Rehabilitation Professionals.

Expert Role	How They Use the Classification
Physiatrists (Physical Medicine and Rehabilitation Physicians)	They interpret the stability levels to guide overall rehabilitation planning, adjust medical management, and coordinate the rehabilitation team.
Physical Therapists	They use the classifications to design balance-training protocols, gait re-education, and fall-prevention programs, scaling exercise intensity according to the patient’s stability level.
Occupational Therapists	They integrate the stability ratings into ADL (activities of daily living) training, ensuring that tasks such as reaching or transferring are matched to the patient’s current stability status.
Rehabilitation Engineers/Assistive-technology Specialists	They embed the gesture-based stability classifier into smart exoskeletons or wearable devices (e.g., EMG-driven gloves) so that mechanical assistance is automatically modulated in real time.
Neurologists and Stroke-specialist Nurses	In post-stroke programs, they monitor stability trends over time and adjust pharmacologic or nursing interventions to minimize fall risk.

## Data Availability

The datasets generated and/or analysed during the current study are available from the third author on reasonable request.
